# Long-term durability and temporal pattern of revisional surgery of laparoscopic large hiatal hernia repair

**DOI:** 10.1007/s13304-025-02070-y

**Published:** 2025-01-23

**Authors:** Elettra Ugliono, Fabrizio Rebecchi, Caterina Franco, Mario Morino

**Affiliations:** 1 Department of Surgical Sciences, General Surgery and Center for Minimally Invasive Surgery, University of Torino, Corso A.M. Dogliotti 14, 10126 Turin, Italy; 2 Department of Mechanical and Aerospacial Engineering, Politecnico of Turin, Corso Duca Degli Abruzzi 24, 10129 Turin, Italy

**Keywords:** Hiatal hernia, Fundoplication, Cruroplasty, Hiatal repair, Recurrence

## Abstract

Laparoscopic repair is the preferred surgical treatment for symptomatic Large Hiatal Hernia (LHH). However, data on long-term outcomes are limited. This study aims to evaluate the 20-year follow-up results of laparoscopic LHH repair in a high-volume experienced tertiary center. Retrospective analysis of patients who underwent elective laparoscopic LHH repair between 1992 and 2008. Preoperative and perioperative data were collected. The primary endpoint was the long-term reoperation rate. Survival analyses were calculated according to the Kaplan–Meier method and compared with the log-rank test. A Cox proportional hazard model was used to investigate predictive factors of the need for revisional surgery. A total of 176 patients were included. All the procedures were performed laparoscopically, and in 5 cases (3.0%) with a robot-assisted approach. Mesh-augmented cruroplasty was performed in 26 patients (15.8%). A fundoplication was added in all patients: Nissen in 158 (89.8%), Toupet in 5 (2.8%), and Collis–Nissen in 13 (7.4%). Postoperative mean follow-up was 224.6 ± 83.3 months. Clinically significant hiatal hernia recurrence occurred in 27 (16.2%), and 18 patients (10.2%) underwent surgical revision. The median time-to-redo was 12 months (IQR 6–42 months). Overall durability without revisional surgery at 20-year follow-up was 90%. The rate of revisional surgery after LHH repair is low and is generally required within 12 months from primary surgery. Our results highlight the long-lasting effects of LHH repair at 20-year follow-up.

## Introduction

Large hiatal hernias (LHH) are commonly defined as a hiatal defect greater than 5 cm or as a displacement of more than one-third of the stomach into the chest, and represent 5–10% of all hiatal hernias [1, 2].

The surgical repair for symptomatic LHH is recommended to achieve control of symptoms and to avoid the risk of complications such as bleeding, obstruction, strangulation, and perforation [3, 4]. The laparoscopic approach is considered the standard of care for the surgical repair of LHH. In fact, it is associated with shorter hospital stay, faster postoperative recovery, lower perioperative morbidity and lower postoperative pain compared to open procedures [5, 6].

There is an extreme heterogeneity of data in the literature regarding the outcomes of laparoscopic LHH repair, with reported rates of postoperative recurrent hiatal hernia ranging from 20 to 50% [7]. Despite these high recurrence rates, several authors previously demonstrated no evidence of a direct correlation between anatomical recurrences and clinical outcomes of patients after laparoscopic LHH repair. Furthermore, revisional surgery was required only in a minority of patients [7]. Since the clinical impact of anatomical recurrences has been questioned, other clinical parameters, such as the need for revisional surgery, have been advocated as the most useful endpoints for the assessment of laparoscopic LHH repair [8].

To date, there is limited information about the long-term follow-up of laparoscopic LHH repair [9–11]. Therefore, the aim of our study is to evaluate the long-term results of a cohort of patients submitted to laparoscopic LHH repair in a high-volume experienced tertiary center.

## Methods

We retrospectively analyzed a cohort of patients who underwent elective laparoscopic LHH repair at the Minimally Invasive Surgery Center of the University of Turin between January 1992 and December 2008.

The inclusion criteria were the presence of symptomatic LHH repaired with a laparoscopic approach. LHH was defined as the translocation of at least one-third of the stomach into the chest, and we used as a more precise threshold an axial herniation of at least 5 cm of the stomach in the chest on the preoperative barium esophagogram. The type of hiatal hernia was defined according to the Skinner and Belsey classification: sliding (type I), true paraesophageal (type II), mixed (type III), and upside-down stomach (type IV). Exclusion criteria were patients who underwent emergent surgical repair or operated with an open approach [12].

A prospective database was retrospectively enquired for preoperative symptoms, instrumental findings, and perioperative data. The severity and frequency of reflux symptoms, and the presence of obstructive, respiratory, cardiologic symptoms and anemia were investigated. Preoperative diagnostic examinations, including upper endoscopy, contrast-medium radiological series, computed tomography (CT) scan.

A standardized operative surgical technique was performed in all patients. Briefly, a 12 mmHg pneumoperitoneum was established, and a standard five-trocar technique was used as previously described [13]. The essential technical steps of the surgical procedure were complete abdominal reduction of LHH by gentle traction, hernia sac dissection and excision, extensive mediastinal mobilization of the esophagus, and tension-free crural closure [14]. In case of a wide diaphragmatic defect (more than 5 cm), in which excessive tension would be applied on the crura with a suture-only cruroplasty, a mesh was added to act as a bridge to cover the gap of an incomplete crural closure. The diaphragmatic crura were approximated posteriorly to the esophagus with interrupted, nonabsorbable sutures until the diaphragmatic muscle fibers can withstand the tension, without attempting to completely cover the diaphragmatic defect with sutures under excessive traction; subsequently, a U-shaped mesh was positioned to cover the suture and the incomplete crural closure, and fixed to the edges of the diaphragmatic defect. The mesh positioned was intended to be definitive.

At the end of the procedure, partial or total fundoplication was routinely added [15]. Intraoperative endoscopy was performed in all patients to identify the location of the esophagogastric junction precisely. In case of insufficient intra-abdominal esophageal length after extensive mediastinal dissection, a Collis–Nissen gastroplasty was performed.

Data on the type of fundoplication, the use of a prosthetic mesh, and additional procedures for short esophagus lengthening were collected. Furthermore, data regarding intra- and postoperative complications and length of stay were evaluated.

The follow-up protocol of our Institution included clinical visits at 1, 6, 12 months, and annually thereafter. In the absence of relevant clinical symptoms, no instrumental examinations were routinely performed after the surgical procedure. In case of suspected clinical recurrence of LHH, procedure-related or mesh-related complications, patients were submitted to the appropriate postoperative investigations (upper endoscopy, contrast-medium radiological series, CT scan). If confirmed, revisional surgery was offered to patients.

### Statistical analysis

The primary endpoint was the long-term reoperation rate of patients who underwent laparoscopic LHH repair. Secondary endpoint was the rate of symptomatic long-term hernia recurrence.

Continuous variables were reported as mean ± standard deviation (DS) when normally distributed and median (Interquartile Range (IQR)) if not, while categorical data were expressed as percentages.

For time-to-event analyses, patients were considered entering the study at the time of the surgical procedure. Follow-up time was considered for each patient until the occurrence of the event of interest or until the last clinical evaluation in the absence of the event of interest. Survival analyses were calculated according to the Kaplan–Meier method and compared with the log-rank test. A Cox proportional hazard model was used to investigate predictive factors of the need for revisional surgery. Variables found significant in univariate analyses were included in a multivariable analysis, and a significance level of 0.15 was required for the variable to remain in the model. A two-tailed p value < 0.05 was considered statistically significant. All the statistical analyses were performed using STATA software version 18.0.

## Results

A total of 176 patients underwent laparoscopic LHH repair at our Institution from January 1992 to December 2008. The mean age at the surgical procedure was 53.7 ± 13.8 years, 74 (42.1%) were males, and 102 (57.9%) were females.

Daily heartburn and regurgitation were reported by 163 (92.6%), obstructive symptoms and epigastric pain were experienced by 91 (51.7%) and 69 (39.2%) patients, respectively. Respiratory symptoms affected 54 (30.7%) patients, while cardiac symptoms (11 patients, 6.2%) and anemia (7 patients, 4.0%) were less frequent.

Preoperatively, all patients underwent upper endoscopy and contrast-medium radiological series, and the instrumental findings are reported in Table 1.

**Table 1:** Preoperative instrumental findings

	N = 176
*Endoscopic findings*	
Grade A esophagitis	43 (24.4%)
Grade B esophagitis	21 (11.9%)
Barrett’s metaplasia	11 (6.2%)
Peptic ulcer	13 (7.4%)
*Radiological contrast-medium series*	
Type I	83 (47.2%)
Type II	2 (1.1%)
Type III	71 (40.3%)
Type IV	20 (11.4%)

All the procedures were performed laparoscopically, and in 5 cases (3.0%) the operation was performed with a robot-assisted approach. Conversion to open surgery was required in 3 (1.8%) patients due to adhesions secondary to previous surgical procedures. Mesh-augmented cruroplasty was performed in 26 patients (15.8%) with different prosthetic materials: a Polypropylene mesh in 14, a Gore-Tex mesh in 7, and a mixed mesh in 5 patients. A fundoplication was added in all patients after hiatal closure: Nissen fundoplication in 158 (89.8%) and Toupet in 5 (2.8%). In 13 patients (7.4%) there was an intraoperative endoscopic diagnosis of short esophagus; consequently, a Collis–Nissen fundoplication was performed.

Intraoperative complications occurred in 3 (1.7%) patients (two spleen injuries and one gastric laceration) and were diagnosed and repaired during the surgical procedure. Perioperative morbidity was 6.6% (bleeding in 5 cases and pneumological complications in 6 cases). There was no intraoperative or postoperative mortality. The mean length of stay was 4.2 ± 2.8 days.

### Follow-up data

Postoperative median follow-up was 243.5 months (IQR 197.5–274.5 months). Instrumental examinations were performed in 80 (45.4%) patients; clinically significant hiatal hernia recurrence occurred in 27 (16.2%), while no mesh-related complications were documented. Among the other 53 patients who underwent instrumental examinations, esophagitis (16 patients), erosive gastritis (7 patients), and regular surgical outcomes (30 patients) were found. A total of 18 patients (10.2%) underwent surgical revision for symptomatic hiatal hernia recurrence, and the median time-to-redo was 12 months (IQR 6–42 months).

**Fig. 1:**
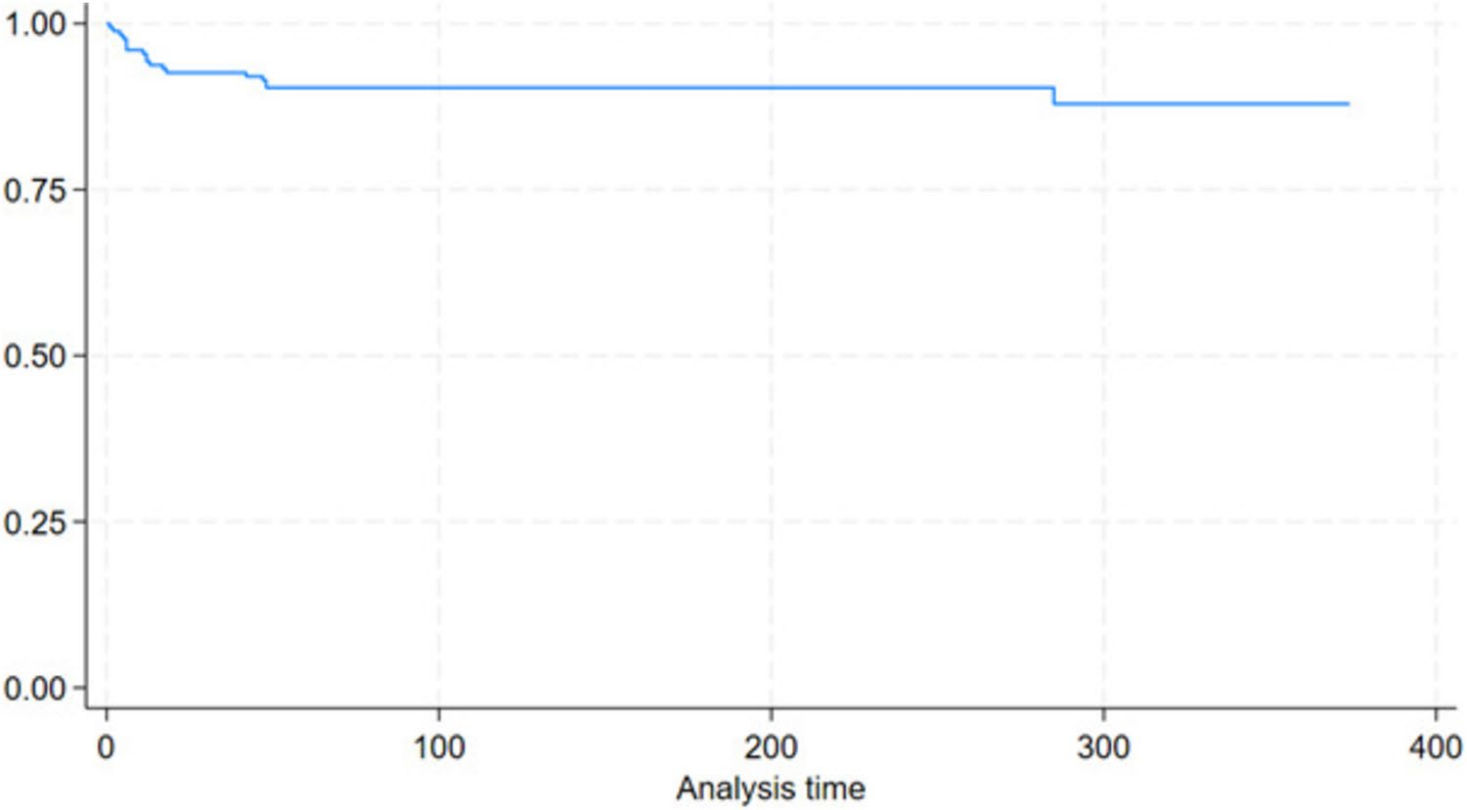
Kaplan–Meier survival curve for redo surgery

Figure 1 shows the Kaplan–Meier curve for redo surgery. Overall durability without need for revisional surgery at 20-year follow-up was 90%. Table 2 shows the overall survival function for redo surgery.

**Table 2:** Overall survival function for redo surgery

Time (months)	Total (at risk)	Redo	Survival function	95% CI
6	172	7	0.96	0.91–0.98
12	168	3	0.94	0.89–0.96
24	164	3	0.92	0.87–0.95
48	161	4	0.90	0.84–0.93
100	157	0	0.90	0.84–0.93
200	131	0	0.90	0.84–0.93
250	85	0	0.90	0.84–0.93
300	20	1	0.87	0.79–0.92
350	3	0	0.87	0.79–0.92

**Fig. 2:**
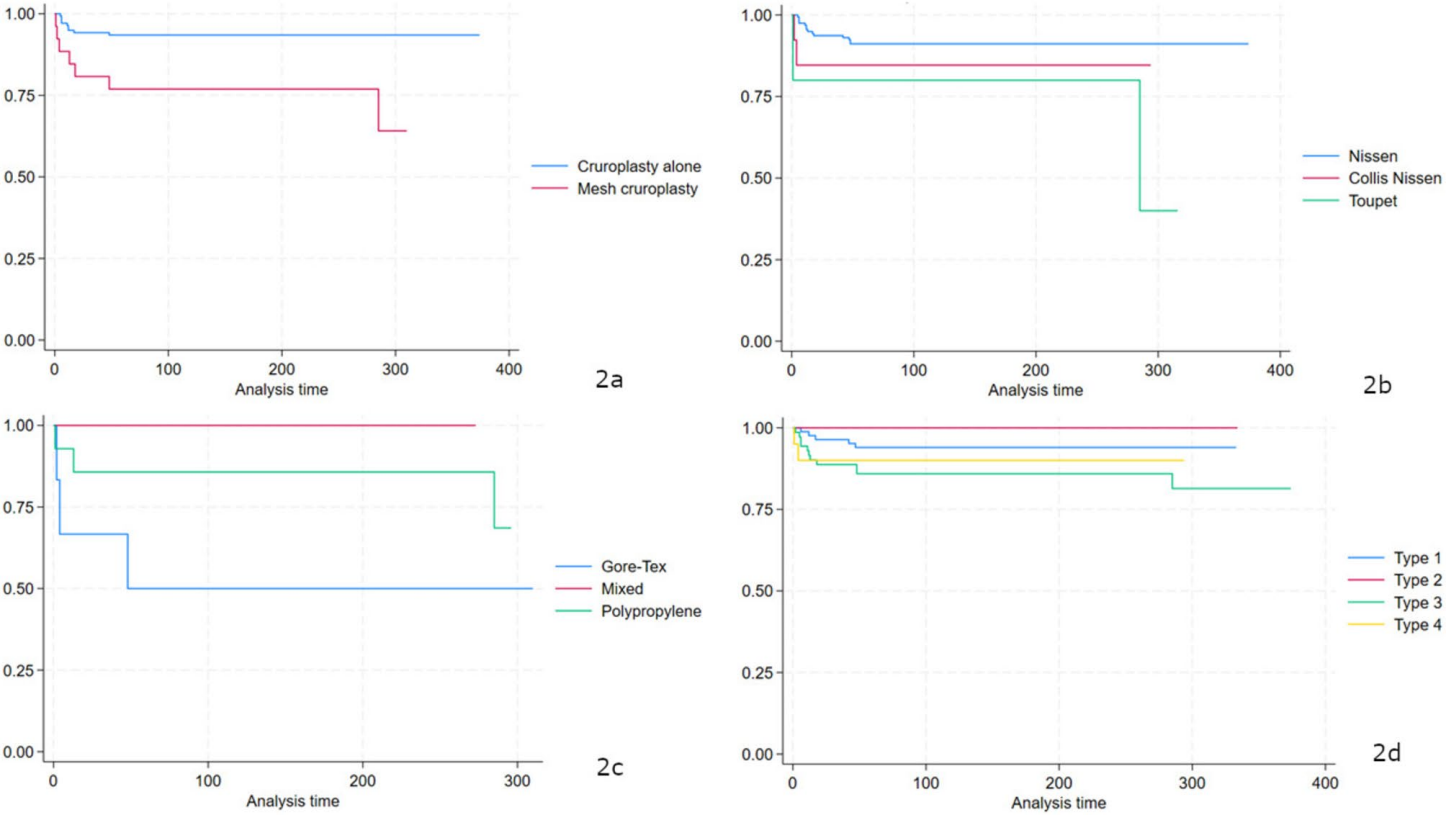
Overall survival depending on technical considerations: the addition of a mesh for crural repair (**a**), the type of wrap (**b**), the type of mesh (**c**) and the type of hiatal hernia (**d**)

The Kaplan–Meier plots reported in Fig. 2 represent the durabilitycurves without revisional surgery depending on technical considerations. Figure 2a shows the overall durability of patients depending on the addition of a mesh for crural repair. Patients who underwent mesh cruroplasty had worse results compared to hiatoplasty alone (p = 0.0012). Furthermore, patients who underwent partial fundoplication had worse results than other fundoplication types (Fig. 2b, p = 0.048). However, there were no differences in overall durability depending on the type of mesh (Fig. 2c, p = 0.18) and on the type of hiatal hernia (Fig. 2d, p = 0.289).

Variables that were included in the multivariable Cox proportional model were type of hiatal hernia (HR 1.39, 95% CI 0.91–2.12, p = 0.11), older age at surgery (HR 1.04, 95% CI 1.0–1.08, p = 0.02), gender (HR 2.70, 95% CI 0.88–8.24, p = 0.08), mesh cruroplasty (HR 4.42, 95% CI 1.64–11.9, p = 0.0059) and the type of wrap (HR 2.20, 95% CI 1.09–4.44, p = 0.028). By multivariable analysis, mesh cruroplasty was a statistically significant predictive factor of the need for revisional surgery (HR 4.33, 95% CI 1.61–11.6, p = 0.006). The results are summarized in Table 3.

**Table 3:** Multivariate analyses according to Cox proportional model

Variable	Complete model			Reduced model		
	Hazard rate	95% CI	p	Hazard rate	95% CI	P
Type of LHH	1.14	0.33–3.98	0.82			
Age at surgery	1.01	0.97–1.07	0.43			
Gender	1.67	0.51–5.47	0.39			
Mesh cruroplasty	3.01	0.90–10.1	0.073	4.33	1.61–11.6	**0.0066**
Type of Wrap	1.50	0.66–3.38	0.32			
Size of HH	0.81	0.12–5.39	0.83			

Bold indicates statistically significant

## Discussion

Laparoscopic surgical repair is considered the treatment of choice for symptomatic LHH [6]. However, the long-term durability of laparoscopic LHH repair is still a matter of debate since there is a paucity of published data regarding long-term clinical and anatomical outcomes. Furthermore, despite wide observation periods reported by the available literature, the mean follow-up time of single patients rarely exceeds 5 years [9–11].

For instance, Blake et al. reported the patient-reported outcomes of 235 patients who underwent LHH repair between 2004 and 2016. The results of the clinical questionnaire administered up to 11 years postoperatively showed significant and lasting symptom improvement, with more than 85% of patients reporting excellent satisfaction at all time points. However, objective follow-up was available only one year after surgery, showing that 8.7% and 2.4% of patients had < 2 cm and > 2 cm hiatal hernia recurrence rates, respectively [16].

Also, La Page et al. reviewed a prospectively maintained database of 455 patients submitted to LHH repair without mesh from 1991 to 2012, with open and laparoscopic approaches, at a single tertiary referral center in Australia. The median follow-up was 32 months (range 0–235 months), the overall reported recurrence rate was 35.6% at a mean of 42 months after surgery, and revision operations were performed in 4.8% of the study population. However, the favorable results of surgery on symptom control were not affected by the presence of a recurrence [17].

These results are consistent with those reported by other authors, showing no direct correlation between the presence of radiological recurrences and worse clinical outcomes of LHH repair [18, 19]. In fact, recurrences after LHH repair are relatively common [2]. According to a meta-analysis performed by Rathore et al., including 13 retrospective studies on the surgical outcomes of laparoscopic LHH repair, the “true” incidence of hiatal hernia recurrence detected with barium esophagogram was 25.5%. However, when considering symptomatic recurrence, the rate decreases to 14%. Furthermore, only 5% of patients required reoperative surgery after LHH repair [7]. Therefore, more clinically significant outcomes, such as the need for revisional surgery, have been proposed as the most valuable endpoints for the objective assessment of laparoscopic LHH repair [8].

In this study, we reported a long-term symptomatic hernia recurrence rate of 16.2% and a reoperation rate of 10.2%, which compares favorably with previously published series [20, 21]. The median time-to-redo was 12 months, meaning that most of symptomatic recurrences requiring revisional surgery occurred within the first year after surgery.

Recurrent hiatal hernias remain a major concern after laparoscopic LHH repair. We strongly adhere to some essential technical surgical principles in an attempt to reduce the rate of postoperative recurrences, such as extensive mediastinal dissection, hernia sac excision, tension-free hiatoplasty, and the addition of a fundoplication [14]. Depending on intraoperative findings, we adopted a selective approach to mesh cruroplasty and esophageal lengthening procedures for short esophagus.

The addition of prosthetic materials for crural repair is still a matter of debate. On the one hand, short-term results of randomized clinical trials have demonstrated that mesh-augmented cruroplasty was associated with a reduced recurrence rate compared to cruroplasty alone [22–24]. However, discordant results were reported in later studies, and controversies remained regarding the optimal shape, size, material, and fixation technique of prosthetic reinforcement [25, 26]. Furthermore, mesh-related complications, whose true incidence might be underreported in the literature, could lead to serious consequences that might outweigh the reduced risk of recurrences [27].

More recently, Petric et al. performed a systematic review and meta-analysis, including seven randomized clinical trials, that compare sutured vs. mesh-augmented cruroplasty, showing no significant differences between the two techniques in terms of patient satisfaction and rate of recurrences, both in the short-term (10.1% mesh vs. 15.5% sutured, p = 0.22) and in the long-term (30.7% mesh vs. 31.3% sutured, p = 0.69) follow-up [28]. Similar results have been reported by Angeramo et al. in another recent systematic review and meta-analysis, which also highlighted the higher overall morbidity associated with non-absorbable mesh (RR 1.45, 95% CI 1.24–1.71, P < 0.01) [29].

In our series, 26 patients (15.8%) underwent mesh hiatal repair, both with absorbable and non-absorbable materials, and we did not experience any mesh-related complications. In this study, we adopted a selected approach to mesh cruroplasty, depending on the size of the hiatal defect evaluated intraoperatively. In the case of a wide hiatal opening, where a primary suture would lead to the tearing of diaphragmatic muscle fibers due to excessive tension, mesh-augmented cruroplasty was performed as a bridge to cover the gap of an incomplete crural closure. This choice could explain the worse results associated with mesh cruroplasty compared to hiatoplasty alone, which we have found in our study; in fact, meshes were added in more complex cases, at higher risk of recurrence due weakened diaphragmatic pillars.

Controversy still exists regarding the optimal surgical approach for the management of LHH. The need to add a fundoplication during LHH repair is still a matter of debate; according to a recent meta-analysis including 22 studies for a total of 8600 patients, there was a trend toward a higher rate of GERD, hernia recurrence, and reoperation when fundoplication was not performed, but a lower risk of dysphagia; however, these data did not reach statistical significance [30]. There is no consensus on the optimal type of wrap; the most commonly performed are Nissen, Toupet of Dor [31]. In our study, five patients had ineffective esophageal motility on preoperative manometry and underwent Toupet fundoplication in order to reduce the mechanical obstacle to the passage of the food bolus offered by the partial posterior wrap. At univariate analysis, Toupet fundoplication appeared to be associated with worse outcomes than Nissen fundoplication; however, these results were not confirmed at multivariable analysis.

The strengths of our study are the large number of LHH patients and the long follow-up period for each patient. However, our study has limitations; first, its retrospective nature. Second, some patients could have experienced a symptomatic recurrence and been reoperated elsewhere. However, this is an unlikely scenario since our Institution is a referral center for complex surgery such as primary and revisional LHH repair. Finally, only symptomatic patients underwent instrumental examination; therefore, the exact rate of recurrences could have been underestimated in this study. However, the overall recurrence rate (symptomatic and asymptomatic recurrences) was not the primary outcome of our study, the presence of small asymptomatic recurrent hiatal hernias has no clinical significance, and the lack of need for instrumental examination confirms the good clinical results of LHH repair at very long follow-up.

## Conclusions

This study demonstrates that the rate of revisional surgery of LHH repair at long-term follow-up is low and is generally required within 12 months from primary surgery. Our results highlight the long-lasting effects of LHH repair, confirming the reliability and effectiveness of this procedure when performed in an experienced surgical center.

## Data Availability

The data that support the findings of this study are available from the corresponding author upon reasonable request.
